# Development and Validation of a Chinese Version of a School-to-Work Transition Anxiety Scale for Healthcare Students

**DOI:** 10.3390/ijerph18147658

**Published:** 2021-07-19

**Authors:** Tzu-Yun Hung, Hung-Chang Liao, Ya-huei Wang

**Affiliations:** 1 Department of Counseling and Applied Psychology, National Taichung University of Education, Taichung 40201, Taiwan; hll7318@gmail.com; 2 Department of Health Policy and Management, Chung Shan Medical University, Taichung 40201, Taiwan; 3 Department of Medical Management, Chung Shan Medical University Hospital, Taichung 40201, Taiwan; 4 Department of Applied Foreign Languages, Chung Shan Medical University, Taichung 40201, Taiwan; 5 Department of Medical Education, Chung Shan Medical University Hospital, Taichung 40201, Taiwan

**Keywords:** school-to-work transition anxiety, healthcare students, scale development, exploratory factor analysis, confirmatory factor analysis

## Abstract

Objective: The aim of this paper was to develop an appropriate scale measuring healthcare students’ anxiety during the transition from school to work. Methods: After an extensive literature review and panel discussion to prove the face validity and content validity, the initial item pool was reduced to 52 items. In a pilot study, a sample of four hundred and twenty-four healthcare students participated, and exploratory factor analysis (EFA) and confirmatory factor analysis (CFA) were used. Psychometric properties—construct validity, convergent validity, discriminant validity, goodness of fit, and reliabilities—were also analyzed. Results: After the use of EFA, the 52 items were reduced to 31 items in four factors, with 66.70% of the total variance explained. The Cronbach’s alpha values ranged between 0.91 and 0.93. The study also used CFA to validate the EFA model, and the results demonstrated that with the same thirty-one items in a 7-point Likert scale, the model was a better fit in four factors: “inexperience in professional knowledge and skills” (nine items; factor loadings: 0.642–0.867; 43.72% of the variance explained), “fear of death” (eight items; factor loadings: 0.745–0.831; 9.94% of the variance explained), “fear of being infected” (eight items; factor loadings: 0.678–0.866; 7.86% of the variance explained), and “interpersonal interactions” (six items; factor loadings: 0.704–0.913; 5.18% of the variance explained). The CFA model demonstrated a good model fit in the χ^2^/df ratio (1.17; *p* = 0.016), CFI (0.99), TFI (0.99), and RMSEA (0.02). The composite reliabilities ranged from 0.89 to 0.92, confirming the StWTA-HS scale’s stability and internal consistency. The convergent validity and discriminant validity were also confirmed. The StWTA-HS scale has been proven to be a stable scale to measure healthcare students’ school-to-work transition anxiety.

## 1. Introduction

The school-to-work transition is a crucial period for students, as they must integrate their professional knowledge, skills, and attitudes into their workplace; it is the period in which students acquire clinical skills, associate with coworkers, and adapt to an unfamiliar healthcare environment. This transition can be defined as a period of adaptation from the acquisition of professional education and training to planning for entry into the workplace and preparation for a career [1]. For healthcare students, Bjerknes and Bjork [2] defined the school-to-work transition as a learning and adjusting process and experience in which students acquire the healthcare knowledge and skills necessary for preparation for their profession and adaptation to the culture and norms of the healthcare workplace. However, while preparing for the multiple competencies required for their careers, young people must also cope with an unpredictable environment once they step into the workplace [3].

Those who cannot make a smooth school-to-work transition may experience vulnerability, lack confidence in their professional knowledge-to-skill performance, and have difficulty maintaining a positive interpersonal relationship with their peers. Moreover, having too much anxiety toward their workplace may lower their efficiency and trigger negative consequences related to their health, family, and social life. In addition, they might express their frustrations and make risky choices in their work environment when they have not yet established themselves [4,5,6]. In essence, if not well prepared for the transition, youth healthcare professionals are also more likely to experience school-to-work transition anxiety and depression, leading to a poorer state of well-being and social cohesion in their healthcare workplace [7].

In the transition to practicing healthcare professions, students have to move from their comfort zone as a student to a stressful healthcare environment where they are expected to rapidly serve as competent healthcare professionals [4]. Hence, in the transition to being a practicing healthcare worker, they might experience anxiety, thinking that they are not skilled in their healthcare profession, which would cause them to lack confidence in their abilities. Healthcare workers have a demanding responsibility to take care of patients. Thus, new workers may be afraid that the absence of practical expertise may lead to unsound judgments, medical errors, or even danger to patients [8]. Moreover, when encountering patients and their families, as well as coworkers, they may not know how to communicate and interact with them or how to handle their questions and demands [8,9,10].

Those not fully prepared for that critical demand in the healthcare workforce tend to experience anxiety, afraid that they are not competent enough to undertake the responsibilities required in the healthcare industry [11]. Moreover, the anxiety of being infected also causes them worry about exposure to viruses at work and the possibility that they might bring the infection home to their families. Especially nowadays, following the outbreak of COVID-19, healthcare workers are at a higher risk of virus contraction. With the rapid increase of new COVID-19-infected cases, these healthcare students are experiencing greater anxiety about being infected, afraid that, by working on the front line, in direct contact with patients who are either confirmed or suspected of having COVID-19, they may become infected themselves [12]. Consequently, they fear that their own exposure to the virus may lead to their families and loved ones being exposed to it.

In addition to the anxiety of being infected, healthcare professionals also experience death anxiety. Junior interns may experience anxiety while talking to a dying patient [13,14]. When dealing with critically ill patients, they experience more anxiety not only because the healthcare responsibility is much more demanding but also because they are forced to face the tension of life-threatening situations on a daily basis. Moreover, for healthcare professionals, death is not only the passing away of a patient; it also signifies the failure or impotence of their clinical or healthcare practice. Therefore, they have more anxiety and stress when encountering the death of a patient [15].

School-to-work transition anxiety has shown significant implications for healthcare students’ social development. Thus, if healthcare students can successfully manage to progress through all the difficult stages in their school-to-work transition, they would build more confidence and professionalism, leading to a stronger self-identity [16]. Therefore, they should raise their awareness in order to conquer their anxiety of facing patient deaths and being infected, perhaps by recognizing that it is extremely common for healthcare students, as well as professionals, to experience anxiety. If this issue is not well addressed, it can pose dangers for those involved.

In recent years, clinical and social researchers have begun to investigate students’ anxiety in transitioning to work. However, despite some research regarding medical students’ and nursing students’ general anxiety [17,18,19], little research has pertained to the anxiety of other healthcare students, much less their school-to-work transition anxiety. In addition, there is no transition anxiety scale based on Taiwanese cultural and medical contexts to measure medical university students’ transition anxiety. Hence, this study was intended to develop a school-to-work transition anxiety scale, which could be used to help healthcare students manage their school-to-work transition anxiety and develop appropriate intervention strategies to use in the healthcare workplace.

## 2. Methodology

### 2.1. Procedure and Participants

The School-to-Work Transition Anxiety scale for Healthcare Students (StWTA-HS) was developed based on five steps. First, a systematic literature review was conducted for the generation of the initial item pool. An expert review panel led to the deletion of inappropriate scale items and the confirmation of the face and content validity of the initial scale items. Then, exploratory factor analysis was used to extract factors and reduce the number of variables. After that, confirmatory factor analysis confirmed the EFA model. Lastly, the assessment of internal consistency and fit of goodness were conducted, which was followed by an examination of the validities and reliabilities.

After conducting the aforementioned series of literature reviews and expert panel discussions on school-to-work transition anxiety regarding medical care and health care, the panel decided to use a Likert scale to develop the School-to-Work Transition Anxiety scale for Healthcare Students (StWTA-HS), because the Likert scale is the most popular behavioral measurement tool [20]. Initially, 60 scale items were collected and scored using a 7-point Likert scale because seven points provided participants with a wider range of response options [21,22], i.e., 7 = strongly agree, 6 = agree, 5 = somewhat agree, 4 = neither agree nor disagree, 3 = somewhat disagree, 2 = disagree, and 1 = strongly disagree. The higher the score is, the stronger the participant’s school-to-work transition anxiety.

The StWTA-HS scale was initially developed in English, later translated into Mandarin Chinese, and then translated back into English. Two bilingual English teachers and two researchers reviewed the editions to ensure that all the scale items were clear, without any ambiguities. In an attempt to prove the face validity and content validity, after collecting the item pool, the study convened experts in social sciences, statistics, and scale assessments in order to examine the validity of the scale [22]. After the expert assessment, eight items were deleted because an agreement was not reached on these items in their relevance to the theoretical construct [23]. After dropping the eight items, the panel sorted the remaining 52 items into four categories: “inexperience in professional knowledge and skills”, “fear of death”, “fear of being infected”, and “interpersonal interactions”.

The initial StWTA-HS scale was administered to four hundred and twenty-four (*N* = 424) randomly selected healthcare students in Taiwan, following the guidelines of the Declaration of Helsinki. The study protocol was approved by the ethics and review committee of Chung Shang Medical University Hospital (No. CS18216). During the data collection, the nature and purpose of the study were explained; anonymity and confidentiality of identities were also ensured.

### 2.2. Data Analysis

The data analysis was composed of EFA (exploratory factor analysis) and CFA (confirmatory factor analysis). EFA, using SPSS (version 14.0) (IBM Corp, Armonk, NY, USA) [24], was used to determine the underlying factor structure and the interrelationships between the factors and the constituent items. After conducting EFA and a principal component analysis, CFA was applied, using AMOS 24.0 (IBM, New York, NY, USA) [25], to validate the internal consistency of the StWTA-HS scale. Psychometric properties—construct validity, convergent validity, discriminant validity, goodness of fit, and reliabilities—were also analyzed.

## 3. Results

### 3.1. Using Exploratory Factor Analysis (EFA) to Identify the Underlying Construct

EFA was used to determine the underlying structure. In addition, a promax rotation, an oblique rotation method allowing intercorrelations between factors, was used to minimize the number of factors [26].

#### 3.1.1. KMO Test and Bartlett’s Test of Sphericity

**Figure 1:**
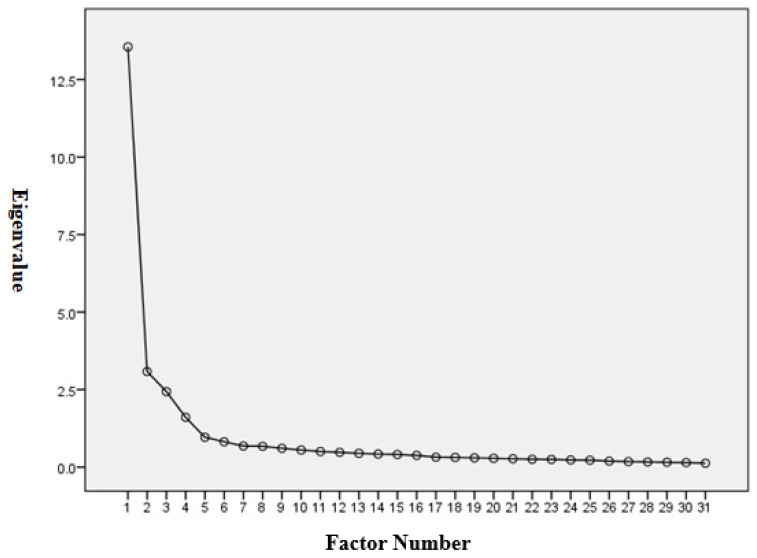
Principal component scree plot of StWTA-HS’s factor structure.

The Kaiser–Meyer–Olkin (KIMO) value of this study was 0.942, which is higher than the cutoff value of 0.6 [27], and Bartlett’s test of sphericity [28,29] reached statistical significance (approximately 9315.425, *p* = 0.000 < 0.05). The KMO’s and Bartlett’s test results proved the appropriateness of the sample size for the EFA model. The scree-plot graphic, with the factor number on the *x*-axis and eigenvalues on the *y*-axis, clearly indicated that four is the optimal number of factors for the StWTA-HS scale (Figure 1).

#### 3.1.2. EFA for Principal Components Analysis

Exploratory factor analysis was conducted to test the construct validity and the internal consistency of the StWTA-HS, using eigenvalues of 1.0 and the promax rotation for principal component analysis [30]. An item was retained if it loaded greater than 0.60 for the relevant factor and less than 0.60 for the nonrelevant factor. Based on the expert discussions on school-to-work transition anxiety for healthcare students, the number of pool items was reduced to 52 for the StWTA-HS on a seven-point Likert scale. After EFA by using a promax rotation for principal components analysis, 31 items and four factors were identified, with 66.70% of the total variance explained: “inexperience in professional knowledge and skills”, “fear of death”, “fear of being infected”, and “interpersonal interactions”. Factor 1 contained nine items related to “inexperience in professional knowledge and skills”, such as “My pressure comes from a lack of professional knowledge and skills”; this factor accounted for 43.72% of the variance explained. Factor 2 contained eight items related to “fear of death”, such as “I feel anxious when thinking of the possibility of facing patient deaths in the future”, and it accounted for 9.94% of the variance. Factor 3 contained eight items related to “fear of being infected”, such as “The possibility of being infected makes me hesitate to be a healthcare provider”, and it accounted for 7.86% of the variance. Lastly, Factor 4 contained six items related to “interpersonal interactions”, such as “Having poor communication skills, I am afraid of facing patients and patient families”, accounting for 5.18% of the variance. The eigenvalues of the four factors from principal component analysis were all larger than 1: 13.55, 3.08, 2.44, and 1.61 (Table 1), respectively, meeting Kaiser’s [31] eigenvalue-greater-than-one rule.

#### 3.1.3. Validity and Reliability of the EFA Model for the StWTA-HS Scale

After deriving the EFA model, Cronbach’s alpha, the most common reliability measure to test the stability and internal consistency of the scale, was used to test the internal consistency of the StWTA-HS, with the alpha coefficient 0.70 as the cutoff value; the higher the alpha coefficient, the higher the internal consistency [32]. As shown in Table 2, the alpha coefficients for the four subscales were 0.93, 0.92, 0.92, and 0.91 in “inexperience in professional knowledge and skills”, “fear of death”, “fear of being infected”, and “interpersonal interactions”, respectively; the alpha coefficient for the entire scale was 0.96. As all the alpha coefficients are higher than the cutoff alpha coefficient of 0.70, the StWTA-HS scale has been proven to be a stable scale to measure healthcare students’ school-to-work transition anxiety.

#### 3.1.4. Scale Item Descriptions, Average Item Scores, and Standard Deviations

The StWTA-HS scale’s item statements, average item scores, standard deviations, and item-total correlations are shown in Table 2.

### 3.2. Using Confirmatory Factor Analysis (CFA) to Validate the EFA Model

**Figure 2:**
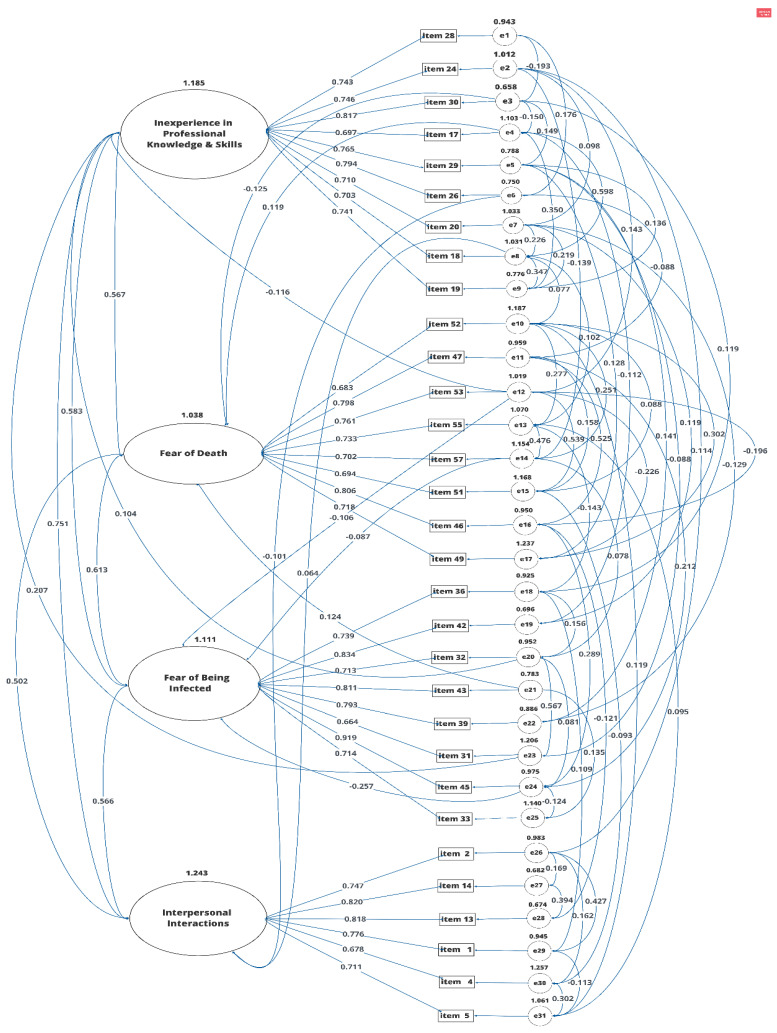
CFA model for the StWTA-HS scale.

After administering the EFA, the 52 items in the StWTA-HS scale were reduced to 31 items in four factors; also, the construct validity and the internal consistency of the scale were confirmed. Then, in order to confirm the developed EFA model, CFA was conducted on the same sample on which the EFA was conducted, using AMOS 24 [25]. As in the EFA model, the four-factor CFA results also yielded the same 31 items, with the same items in each factor: “inexperience in professional knowledge and skills” (nine items; factor loadings: 0.70–0.82), “fear of death” (eight items; factor loadings: 0.68–0.81), “fear of being infected” (eight items; factor loadings: 0.66–0.92), and “interpersonal interactions” (six items; factor loadings: 0.68–0.82). The factor loadings were all above the minimum acceptable factor loading value 0.60 [33]. The four-factor CFA model for the thirty-one items is shown in Figure 2.

#### 3.2.1. Goodness of Fit of the CFA Model for the StWTA-HS Scale

To examine the goodness of fit of the CFA model for the StWTA-HS scale, the χ^2^/df ratio (chi-square divided by the degrees of freedom), CFI (comparative fit index), TLI (Tucker–Lewis index), and RMSEA (root mean square error of approximation) were compared with the EFA model. There has been no consensus regarding an acceptable χ^2^/df ratio. Some research [34,35] has suggested that a ratio under 5 can be considered a reasonable model fit. Indeed, Tabachnick and Fidell [36] suggested that if the ratio is below 5, the model would be considered good. More specifically, Diamantopoulos and Siguaw [37] suggested that the χ^2^/df ratio fall between 2 and 5. However, Schumacker and Lomax [38] suggested that a χ^2^/df ratio lower than 2.0 be regarded as acceptable.

A CFI > 0.9 can be considered acceptable, with the value > 0.95 being regarded as good [39,40]. A TLI > 0.90 is considered an acceptable model fit, and a TLI > 0.95 is considered an excellent model fit [41,42]. RMSEA ≥ 0.1 indicates a poor model fit, RMSEA in the range of 0.08 ≤ RMSEA < 0.1 suggests a mediocre model fit [43], an RMSEA index in the range of 0.05 ≤ RMSEA < 0.08 is regarded as acceptable, and an RMSEA index < 0.05 is regarded as a good model fit [38].

As shown in Table 3, in the EFA model, the χ^2^/df ratio is 4.14 (*p* = 0.000), CFI is 0.86, TLI is 0.85, RMSEA is 0.09, and the CI for RMSEA is (0.08, 0.09). Furthermore, the two variables that had a high covariance were connected in order to modify the indices until they all reached goodness of fit [34,35,36,37,38,39,40,41,42,43]; the CFA model is shown in Figure 2. Regarding the modification indices in the CFA model, the χ^2^/df ratio is 1.17 (*p* = 0.016), CFI is 0.99, TLI is 0.99, RMSEA is 0.02, and the CI for the RMSEA is (0.01, 0.03). Thus, from the EFA model to the CFA model, regarding the improvement of indices, χ^2^/df had a decrease of 2.97 (the *p* value increased from 0.000 to 0.016), the CFI showed an increase of 0.13, the TLI increased by 0.14, and the RMSEA had a decrease of 0.07.

#### 3.2.2. Reliabilities for “Inexperience in Professional Knowledge and Skills”, “Fear of Death”, “Fear of Being Infected”, and “Interpersonal Interactions” Factors

EFA was used to reduce the number of items and determine the underlying variables that contained the statement items; the statistical results showed four factors and thirty-one items. While using confirmatory factor analysis to test the underlying construct of the EFA model, coincidently, there were still four factors with the same thirty-one item statements as in the EFA model. Additionally, the CFA model shares the same Cronbach’s alphas with the EFA model; that is, the Cronbach’s alpha values for the four subscales were 0.93, 0.92, 0.92, and 0.91, respectively, and the Cronbach’s alpha for the entire questionnaire was 0.96. The study further used composite reliabilities (CR) to confirm the scale’s stability and internal consistency. According to Hair et al. [44], the CR value should be at least 0.70; the higher the value is, the better the internal consistency. The derived composite reliability coefficients were 0.92, 0.91, 0.92, and 0.89, respectively, which are all higher than the cutoff value of 0.70. Since the values are all higher than the minimally acceptable value of 0.70, it can be assumed that the finalized StWTA-HS scale demonstrated acceptable to excellent reliabilities in assessing participants’ school-to-work transition anxiety.

#### 3.2.3. Convergent Validity

The researchers used average variance extracted (AVE) values to test the convergent validity of the StWTA-HS scale; the scale would be proven to be in good convergent validity only if the AVE values were equal or greater than 0.50 and were smaller than the composite values [44,45]. As shown in Table 4, the AVE values of the “inexperience in professional knowledge and skills”, “fear of death”, “fear of being infected”, and “interpersonal interactions” are 0.56, 0.55, 0.60, and 0.58, respectively, which are all higher than the cutoff value of 0.50. In addition, all of the AVE values are smaller than their corresponding composite reliabilities: 0.92, 0.91, 0.92, and 0.89. Hence, it can be concluded that the StWTA-HS scale has good convergent validity.

#### 3.2.4. Discriminant Validity

To confirm the discriminant validity of the StWTA-HS scale, the researchers compared the square root values of the AVE (√AVE) with the correlation coefficients (*r*) between factors. Discriminant validity is demonstrated when the √AVE is higher than the correlation coefficients (*r*) between factors [45]. As shown in Table 4, the discriminant validity was proven between the factors of “inexperience in professional knowledge and skills” and “fear of death” (√AVE = 0.75 and 0.74, respectively; *r* = 0.57), of “inexperience in professional knowledge and skills” and “fear of being infected” (√AVE = 0.75 and 0.78, respectively; *r* = 0.58), of “inexperience in professional knowledge and skills” and “interpersonal interactions” (√AVE = 0.75 and 0.76, respectively; *r* = 0.75), of “fear of death” and “fear of being infected” (√AVE = 0.74 and 0.78, respectively; *r* = 0.61), of “fear of death” and “interpersonal interactions” (√AVE = 0.74 and 0.76, respectively; *r* = 0.75), and of “fear of being infected” and “interpersonal interactions” (√AVE = 0.78 and 0.76, respectively; *r* = 0.57). Table 4 shows the AVE, √AVE, and correlation coefficients (*r*) between factors. The StWTA-HS scale demonstrated discriminant validity.

The examination of validities and reliabilities has provided evidence that the StWTA-HS scale (Appendix A) can be a reliable instrument to measure school-to-work transition anxiety for healthcare students.

## 4. Discussion

The purpose of this study was to develop a school-to-work anxiety scale. After a thorough literature review to collect item statements, the study convened panel discussions to screen out weak items and sort the collected items into four categories as a priori hypothetical model, which were “inexperience in professional knowledge and skills”, “fear of death”, “fear of being infected”, and “interpersonal interactions”. Although it was not required to conduct the EFA first in the study, in order to verify the priori hypothetical model, the researchers took a more conservative way, taking the EFA statistic technique first to validate the priori hypothetical model on the dataset and then used the CFA statistical technique to once verify whether the dataset was suitable for the model [46,47].

There is no extraction criterion that was proved to be the best to determine the number of factors [48]. Parallel analysis [49] has been taken as a good method for determining the number of factors; however, research has long questioned its theoretical justification [50,51]. The scree-plot test has been a commonly used method to determine the number of factors [52]; however, when the sample size is small, it may bring ambiguity or no clear break or shift in the graph [53,54]. Though both with their limitations, when the sample size is large enough, most approaches will turn in similar results [55,56]. The study used the scree-plot graphic to determine the number of factors in EFA. However, the limitation may be addressed when the sample size is large and the factors are strong and well-defined [55]. The sample size used in the study was 424, meeting the minimum criterion of sample size set out by Hair et al. of 260 (5 times 52 items) for multivariate data analysis research [44]. According to Hair et al. [44], the sample size should be at least five times the amount of items used in the scale. With an appropriate sample size, the possible subjectivity, ambiguity, and bias in the scree-plot test would be eliminated. In the study, with appropriate sample size and well-defined factors, the scree-plot test (as Figure 1) indicates a clear shift and definite break [53,55], showing that four is the optimal number of factors for the StWTA-HS scale. After EFA by using promax rotation for principal component analysis, 31 factors in four factors were identified, with 66.70% of the total variance explained: “inexperience in professional knowledge and skills” (nine items), “fear of death” (eight items), “fear of being infected” (eight items), and “interpersonal interactions” (six items). The rotated factor matrix for the promax oblique solution, as in Table 1, also shows that there is no cross-factor loading greater than |0.276|, hence fully supporting the four-factor solution [44].

The subscale means showed that the respondents scored the highest on the “inexperience in professional knowledge and skills” subscale (mean = 4.21; 37.85 ÷ 9 = 4.21), which is followed by “fear of death” (mean = 4.09) and “interpersonal interactions” (mean = 3.87). The participants scored the lowest on the “fear of being infected” subscale (mean = 3.55). The subscale mean showed that these respondents were anxious about their insufficient professional knowledge and skills, particularly that they may not be sufficiently qualified to be healthcare professionals. In addition, while thinking about facing the deaths of patients and dealing with death telling to patients and their families, they felt uneasy. They might also feel guilty if they fail to save patients’ lives. Moreover, they were concerned that they did not know how to properly communicate with patients and their families. However, compared with the anxieties regarding insufficient professional knowledge and skills, fear of death, and interpersonal interaction, these respondents were not as afraid of being infected. In the EFA model, the factor loadings ranged from 0.65 to 0.79, which are all greater than the cutoff value of 0.60, hence providing evidence for the construct validity of the StWTA-HS [44].

The researchers further used CFA, using AMOS 6.0, to examine the factorial validity of the EFA model for the StWTA-HS scale [57]. The factor loadings for each item statement ranged between 0.66 and 0.92, indicating that the EFA model is an adequate factor indicator. While using CFA to confirm the EFA model, using modification indices to improve the values of model fitness [58], the indices also demonstrated a good model fit in the CFA model [38,42,59]. For both the EFA and CFA model, the Cronbach’s alphas for the overall StWTA-HS scale and all the subscales were above the threshold of 0.70 [32], and the composite reliabilities for the subscales of the CFA model were also above the cutoff point (0.70) [44,60]. In the CFA model, the factor loadings ranged from 0.66 to 0.92, hence demonstrating the construct validity of the StWTA-HS scale. To confirm the convergent validity, the AVE values of the four factors, “inexperience in professional knowledge and skills”, “fear of death”, “fear of being infected”, and “interpersonal interactions”, respectively, are all above the cutoff point of 0.50 and below the corresponding composite reliability values. Hence, the convergent validity of the StWTA-HS scale was demonstrated.

Discriminant validity was also proven to exist based on the Fornell–Larcker criterion [60] whereby the √AVE should be higher than the correlation coefficients (*r*) between factors. The study provided evidence of discriminant validity between the “inexperience in professional knowledge and skills” and “fear of death” subscales, between “inexperience in professional knowledge and skills” and “fear of being infected”, between “fear of death” and “fear of being infected”, between “fear of death” and “interpersonal interactions”, and between “fear of being infected” and “interpersonal interactions”. As with the discriminant validity between the factors of “inexperience in professional knowledge and skills” and “interpersonal interactions”, the √AVE (0.76) of “interpersonal interactions” is higher than the coefficient (*r* = 0.75); however, the √AVE (0.75) of “inexperience in professional knowledge and skills” is just equal to the coefficient (*r* = 0.75).

Based on the above examination of the psychometric properties, including the goodness-of-fit indices, internal consistency, convergent validities, discriminant validities, and so forth, this study has concluded that the CFA model confirmed the EFA model in terms of satisfactory goodness of fit and relevant validities and reliabilities. The research results have proven satisfactory validities and reliabilities. However, it should be noted that those intending to use the scale to assess students’ anxiety during the transition from school to work should first consider students’ cultural and professional backgrounds. Future study may use Horn’s [50] parallel analysis with ordinal variable in EFA to validate the consistency and the number of factors retained in the StWTA-HS scale.

## 5. Conclusions

This study aimed to develop a scale measuring healthcare students’ school-to-work transition anxiety, first using EFA to derive the factors and relevant items and then using CFA to improve the EFA model. The findings proved that the developed StWTA-HS scale is a reliable and psychometrically appropriate scale measuring anxiety during the transition from school to work.

## Figures and Tables

**Table 1:** Rotated factor loading after the promax rotation for the StWTA-HS.

Item	Factor 1: Inexperience in Professional Knowledge and Skills	Factor 2: Fear of Death	Factor 3: Fear of Being Infected	Factor 4: Interpersonal Interactions
Factor 1: α = 0.93				
20	**0.867**	−0.041	0.048	−0.125
19	**0.863**	0.036	−0.053	−0.003
18	**0.827**	0.047	−0.102	0.059
26	**0.805**	−0.046	0.097	−0.029
29	**0.766**	−0.007	0.028	0.042
17	**0.766**	0.164	−0.181	0.082
30	**0.733**	−0.110	0.239	0.011
28	**0.669**	−0.003	0.152	0.009
24	**0.642**	0.092	−0.039	0.156
Factor 2: α = 0.92				
52	0.032	**0.831**	−0.106	0.038
53	−0.118	**0.811**	0.053	0.018
57	0.084	**0.801**	−0.121	0.036
49	0.047	**0.795**	−0.104	−0.009
47	0.042	**0.790**	0.157	−0.117
51	−0.063	**0.789**	0.087	−0.026
55	0.083	**0.778**	−0.011	−0.021
46.	0.000	**0.745**	0.204	−0.072
Factor 3: α = 0.92				
36	−0.065	−0.004	**0.866**	−0.014
42	−0.004	0.021	**0.826**	0.004
32	0.119	−0.105	**0.819**	−0.012
39	−0.063	0.065	**0.786**	0.029
43	−0.116	0.115	**0.783**	0.086
31	0.276	−0.155	**0.746**	−0.103
33	0.009	0.015	**0.710**	0.072
45	0.001	0.174	**0.678**	0.069
Factor 4: α = 0.91				
2	−0.094	−0.060	0.074	**0.913**
14	0.064	0.049	−0.022	**0.809**
1	−0.107	0.032	0.147	**0.809**
4	0.092	−0.100	−0.033	**0.798**
13	0.099	0.073	−0.048	**0.780**
5	0.150	−0.042	−0.010	**0.704**
Eigenvalue	13.55	3.08	2.44	1.61
% of Variance	43.72	9.94	7.86	5.18
Rotation Sums of Squared Loadings	10.36	8.91	9.49	9.21

Overall α = 0.96; total variance explained is 66.70%; The values shown in bold are the rotated factor loadings of the derived four factors after promax rotation.

**Table 2:** Scale item description, average item scores, standard deviations, and item-total correlations of the EFA on the StWTA-HS.

Item	Mean	*S.D.*	Item-Total Correlation
Factor 1: Inexperience in Professional Knowledge and Skills	37.85	10.19	0.849 **
20. I often feel very frustrated and want to quit my job when thinking of my inexperience with medical/healthcare professionalism.	3.77	1.46	0.628 **
19. I experience pressure when thinking that my professional knowledge and skills are not good enough for caring for patients.	4.50	1.33	0.696 **
18. I feel anxious and stressful when thinking that I am not yet familiar with medical/healthcare equipment, technology, and facilities.	4.37	1.46	0.685 **
26. The unsettled conflicts between the ideals and the reality of the healthcare system cause me to doubt whether I am able to be a qualified medical/healthcare professional.	4.08	1.43	0.685 **
29. My pressure comes from a lack of professional knowledge and skills.	4.30	1.38	0.684 **
17. I experience anxiety when thinking that my lack of medical/healthcare professional knowledge and skills may cause me to make errors or endanger someone.	4.70	1.42	0.681 **
30. Thinking that I am not qualified enough to be a medical/healthcare professional leads me to doubt whether I can be a medical/healthcare worker after graduation.	3.97	1.42	0.720 **
28. After making frequent mistakes, I become afraid of caring for patients or providing any healthcare service.	4.01	1.50	0.682 **
24. I feel exhausted, physically and spiritually, after entering the healthcare workplace and realizing that there is a lot of room for improvement in my healthcare knowledge and skills.	4.21	1.47	0.693 **
Factor 2: Fear of Death	32.75	9.93	0.782 **
52. I feel guilty or cannot even sleep when thinking that I may not be able to save patients’ lives.	4.54	1.49	0.615 **
53. Because of some bad experiences in the past, I am afraid of dealing with death issues.	3.72	1.56	0.586 **
57. Death-telling is a very unpleasant emotional experience; I really don’t know how to deal with it.	4.33	1.50	0.618 **
49. I feel very uncomfortable thinking that someone may die because of my unprofessional healthcare knowledge or skills.	4.57	1.59	0.566**
47. I feel anxious when thinking of the possibility of facing patient deaths in the future.	3.66	1.63	0.678**
51. My past experience in facing death has made me feel uncomfortable and anxious in facing patients’ death.	3.75	1.51	0.608 **
55. I don’t know how to tell patients that they are going to die, and hence I experience anxiety.	4.40	1.52	0.676 **
46. Thinking that I have to face the death of my patients makes me question whether I am ready to be a healthcare professional.	3.81	1.64	0.680 **
Factor 3: Fear of Being Infected	28.37	9.45	0.818 **
36. The risk of being infected renders me unable to demonstrate my specialities and professionalism in caring for patients.	3.25	1.44	0.623 **
42. I am too anxious to sleep when thinking that patients may conceal their medical history.	3.40	1.52	0.675 **
32. The possibility of being infected makes me hesitate to be a healthcare provider.	3.39	1.40	0.660 **
39. Thinking that patients may conceal their medical history makes me uneasy and restless; hence, I fail to concentrate on my healthcare job.	3.87	1.55	0.648 **
43. I become anxious upon thinking of the increasing possibility of being infected in the current healthcare system.	3.73	1.52	0.683 **
31. I constantly feel anxious about the possibility of being infected.	3.42	1.48	0.623**
33. I don’t think Taiwan’s medical system can provide medical personnel with a secure working environment, so I feel uneasy about it.	3.91	1.53	0.643 **
45. I don’t know how to deal with patients with infectious diseases, so, when dealing with them, I feel very nervous, and my heartbeat starts racing.	3.55	1.44	0.726 **
Factor 4: Interpersonal Interactions	23.20	7.36	0.800 **
2. Having poor communication skills, I am afraid of facing patients and patients’ families.	3.73	1.49	0.651 **
14. I experience anxiety upon thinking that I may not be able to communicate effectively with patients and patients’ families.	3.73	1.45	0.705 **
1. Thinking of having to communicate with patients and patients’ families in the future makes me uneasy and restless.	3.81	1.54	0.686 **
4. The interpersonal interactions in the healthcare workplace causes me stress.	3.92	1.53	0.602 **
13. Determining how to interact with patients and patients’ families always brings me anxiety.	3.69	1.43	0.708 **
5. I feel nervous and anxious when I have to face my bosses and my supervisors.	4.29	1.48	0.638 **

*S.D.* = Standard Deviation. ** *p* < 0.01

**Table 3:** Goodness-of-fit indices for the baseline EFA model and the CFA model for the StWTA-HS scale.

	χ^2^/df	*p*	CFI	TLI	RMSEA	90% RMSEA CI
EFA Model	4.14	0.000	0.86	0.85	0.09	[0.08, 0.09]
CFA Model	1.17	0.016	0.99	0.99	0.02	[0.01, 0.03]

Note: *n* = 424; χ^2^ = Chi-square; df = degree of freedom; CFI = comparative fit index; TLI = Tucker–Lewis index; RMSEA = root mean square error of approximation; CI = Confidential Index.

**Table 4:** Average variance extracted (AVE), square root of AVE (√AVE), and correlations between factors.

Factor	AVE	1	2	3	4
1. Inexperience in Professional Knowledge and Skills	0.56	0.75			
2. Fear of Death	0.55	0.57 **	0.74		
3. Fear of Being Infected	0.60	0.58 **	0.61 **	0.78	
4. Interpersonal Interactions	0.58	0.75 **	0.50 **	0.57 **	0.76

The values shown in bold are the square root of AVE (√AVE). ** *p* < 0.001.

## Appendix A. The Factors and Items of CFA Model for the StWTA-HS Scale

**Factor 1:** Inexperience in Professional knowledge and Skills (nine items)

1. I often feel very frustrated and want to give up the job while thinking of my immaturity in medical/healthcare professionalism.

2. I experience pressure while thinking that my professional knowledge and skills are not good enough for caring for patients.

3. I feel anxious and stressful while thinking that I do not yet familiarize myself with medical/healthcare equipment, technology, and facilities.

4. The unsettled conflicts between the ideal and the reality in the healthcare system lead me to begin to doubt about whether I am able to be a qualified medical/healthcare professional.

5. My pressure comes from the lack of professional knowledge and skills.

6. I experience anxiety while thinking that my immaturity in medical/healthcare professional knowledge and skills may lead to me making errors or bring trouble to someone.

7. Thinking that I am not qualified enough to be a medical/healthcare professional makes me begin to think about whether I can be a medical/healthcare worker after graduation.

8. After keeping making mistakes all the time, I become so scared of caring for patients or providing any healthcare service.

9. I feel exhausted: physically and spiritually, after entering the healthcare workplace and realizing that there is a lot of room for improvement in my healthcare knowledge and skills.

**Factor 2:** Fear of Death (eight items)

10. I feel guilty or even fail to fall asleep while thinking that I may not be able to save patients’ lives.

11. Because of some bad experience in the past, I am scared of dealing with death issues.

12. Death telling is a very unpleasant emotional experience; I really don’t know how to deal with it.

13. I feel very uncomfortable once thinking that someone may die because of my unprofessional healthcare knowledge or skills.

14. I feel anxious when thinking of the possibility of facing patient deaths in the future.

15. My past experience in facing death has let me afterwards feel uncomfortable and anxious in facing patients’ deaths.

16. I don’t know how to tell patients that they are going to die, and hence I experience anxiety.

17. Thinking that I have to face the deaths of my patients makes me feel like I am not ready yet to be a healthcare professional.

**Factor 3:** Fear of Being Infected (eight items)

18. The risk of being infected makes me unable to demonstrate my specialities and professionalism in caring for patients.

19. I am too anxious to fall asleep due to thinking that patients may conceal their medical history.

20. The possibility of having the risk of being infected makes me hesitate to be a healthcare provider.

21. Thinking that patients may conceal their medical history makes me uneasy and restless, hence failing to concentrate on my healthcare job.

22. I become anxious once thinking of the increasing possibility of being infected in the current healthcare system.

23. At any moment, I feel anxious about the possibility of being infected.

24. I don’t think Taiwan’s medical system can provide medical personnel with a secured working environment, so I feel uneasy about it.

25. I don’t know how to deal with the patients with infectious diseases, so while dealing with them, I feel very nervous and my heart beats faster.

**Factor 4:** Interpersonal Interactions (six items)

26. Having poor communication skills, I am afraid of facing patients and patients’ families.

27. I experience anxiety once thinking that I may not be able to communicate effectively with patients and patients’ families.

28. Thinking of having to communicate with patients and patients’ families in the future makes me uneasy and restless.

29. The interpersonal interaction in the healthcare workplace is a source of pressure for me.

30. How to interact with patients and patient families always brings me anxiety.

31. I feel nervous and anxious when I have to face my bosses and my supervisors.
